# Systemic and Local Factors’ Influence on the Topological Differences in Deep Vein Thrombosis

**DOI:** 10.3390/medicina55100691

**Published:** 2019-10-16

**Authors:** Ştefan Cristian Vesa, Romeo Chira, Sonia Irina Vlaicu, Sergiu Pașca, Sorin Crișan, Adrian Trifa, Anca Dana Buzoianu

**Affiliations:** 1 Department of Pharmacology, Toxicology and Clinical Pharmacology, “Iuliu Haţieganu” University of Medicine and Pharmacy, 400337 Cluj-Napoca, Romania; stefanvesa@gmail.com (Ş.C.V.); ancabuzoianu@yahoo.com (A.D.B.); 2 Department of Gastroenterology, 1st Medical Clinic, “Iuliu Haţieganu” University of Medicine and Pharmacy, 400006 Cluj-Napoca, Romania; romeochira@yahoo.com; 3 Department of Internal Medicine, 1st Medical Clinic, “Iuliu Haţieganu” University of Medicine and Pharmacy, 400006 Cluj-Napoca, Romania; vlaicus@yahoo.com; 4 Department of Internal Medicine, 5th Medical Clinic, “Iuliu Haţieganu” University of Medicine and Pharmacy, 400139 Cluj-Napoca, Romania; 5 Department of Medical Genetics, “Iuliu Haţieganu” University of Medicine and Pharmacy, 400349 Cluj-Napoca, Romania; crisan.sorin@gmail.com (S.C.); trifa.adrian@gmail.com (A.T.)

**Keywords:** deep vein thrombosis, risk factors, topological localization

## Abstract

*Background and Objectives:* Deep vein thrombosis (DVT) is a common cause of intra-hospital morbidity and mortality, and its most severe complication is pulmonary thromboembolism. The risk factors that influence the apparition of DVT are generally derived from Virchow’s triad. Since the most severe complications of DVT occur in proximal rather than distal deep vein thrombosis, the aim of this study was to identify the factors influencing the apparition of proximal DVT. *Materials and Methods:* This was a transversal, cohort study. The study included 167 consecutive patients with lower limb DVT over a two-year period. The following data were recorded or determined: general data, conditions that are known to influence DVT, medical history and coagulation or thrombophilia-related genetic variations. *Results:* In the univariate analysis, male gender, neoplasia, previous DVT and mutated factor V Leiden were all associated with proximal DVT, while bed rest was associated with distal DVT. In the multivariate analysis, male gender, previous DVT and factor V Leiden mutation were independently correlated with proximal DVT, while bed rest was independently associated with distal deep vein thrombosis. *Conclusion:* Our observations point out that the factors indicating a systemic involvement of coagulation were correlated with proximal DVT, while local factors were associated with distal DVT.

## 1. Introduction

Deep vein thrombosis (DVT) is a common cause of intra-hospital mortality with a reported incidence of 1/1000/year [1,2,3], although some consider this number to be higher, possibly accounting for a high number of deaths by unknown causes [4]. A major complication that occurs following a DVT episode is pulmonary thromboembolism, with a considerable mortality rate, thus inciting significant concern in the medical community [5,6].

Although first described in 1856, Virchow’s triad still stands as a good starting point to determine the risk factors for DVT since it integrates venous stasis, endothelial damage and hypercoagulability [7]. Consequently, the majority of the DVT risk factors known today are related to Virchow’s triad, for example: obesity, prolonged immobilization, surgery, trauma, vasculitis, neoplasms, or predisposing genotypes [8]. Considering the influence of gender, it has been shown that male gender is a predisposing factor for venous thrombosis and, moreover, for proximal rather than for distal DVT [9,10,11]. Other factors, like single nucleotide polymorphisms (SNPs), have not yet been introduced to the routine clinical practice, but have aroused considerable interest in the research community year after year. A plethora of evidence has established the ability of these genetic factors to influence the predisposition for DVT. The genes critical to DVT occurrence are generally involved in coagulation or oxidative stress. Nonetheless, the question remains whether they can be relevant and cost-effective in the current clinical practice [12,13].

From a topological standpoint, lower limb DVT can be classified as distal below the popliteal vein, and proximal from the popliteal vein upwards. This feature is very important, since proximal DVT has a higher risk of thromboembolism when compared with distal DVT [14]. The risk differences have been validated by a consistent number of studies showing that distal DVT generally does not extend proximally and that in patients with this condition, anticoagulants do not influence mortality [15,16,17,18,19,20]. Therefore, the main cause of venous thromboembolism and of pulmonary thromboembolism (PTE) is represented by proximal DVT.

Given the significant prognostic difference that exists between proximal and distal DVT, one might presume that there are several factors that drive two different etiopathogenetic mechanisms, resulting in either proximal or distal DVT. We hypothesized that systemic procoagulant factors such as factor V Leiden mutations or neoplastic disease, which systemically shift the coagulation equilibrium to the procoagulant side, will provoke proximal or extensive DVT. In the case of distal DVT, we hypothesized that it might be influenced mainly by local factors, such as an increase in venous pressure and endothelial damage. The rationale behind this is that any cause of venous insufficiency will determine an increase in pressure starting from the distal to the proximal segment of the vein [6,7].

Considering the aforementioned arguments, the aim of this study was to identify the risk factors that lead to proximal DVT more frequently than to distal DVT.

## 2. Materials and Methods

This was a transversal, observational, prospective, analytical, cohort-type study.

The study included consecutive patients who were diagnosed with acute lower extremity DVT. The patients were selected from those admitted to the Clinical Municipal Hospital of Cluj-Napoca in the time period January 2017–July 2018.

All participants signed the informed consent for the evaluations done in the study. The present study was approved by the Ethics Committee of “Iuliu Hațieganu” University of Medicine and Pharmacy, Cluj-Napoca, Romania (no. 350/13.11.2014) and was performed in accordance with the declaration of Helsinki.

Inclusion criteria were: the diagnosis of acute lower limb DVT, aged over 18 years, and signing the informed consent form.

Exclusion criteria were: age under 18 years, pregnancy, oral contraceptive use, or refusal to sign the informed consent form.

The diagnosis of DVT was established according to the guidelines [21]. Patients with suspected lower limb DVT, with a Wells score of higher than 2, were examined with a venous ultrasound. The examination was performed on all profound (common femoral vein, superficial and profound femoral veins, popliteal vein, posterior and anterior tibial veins, peroneal vein, muscular calf vein) and superficial veins (great and small saphenous veins) of the lower limbs on an Aloka SSD 4000 unit with a linear transducer at a frequency between 7 and 10 MHz. The DVT diagnosis was based on the following criteria: vein incompressibility, enlarged vein with direct thrombus visualization, and abnormal spectral and color-Doppler flow.

The following general data were recorded for each patient: age, gender, place of origin, body mass index, a history of car or plane trips over 4 h, immobilization for over 3 days, bed rest for over 5 days; the presence of conditions that might increase the risk of DVT: neoplasia, chemotherapy, diabetes, superficial vein thrombosis (SVT), varicose veins, infectious diseases, autoimmune disease, fracture, local trauma, heart failure; and medical history: previous DVT, previous PTE, major surgery in the last month.

Peripheral blood was collected in a vacutainer containing EDTA. DNA extraction from the peripheral blood was performed using a genomic DNA purification kit (Wizard Genomic DNA Purification Kit, Promega, Madison, WI, USA). The protocols for determining the polymorphisms of factor V Leiden G1691A [22], Prothrombin G20210A [23], PAI-1 4G/5G [24], MTHFR C677T [9], MTHFR A1298C [25], were described in the referred papers.

Statistical analysis was performed using software R version 3.5.2 (R Foundation for Statistical Computing, Vienna, Austria). Normality of distribution was determined using the Shapiro–Wilk test and histogram visualization. Contingency tables were analyzed using the Fisher test. Non-normally distributed variables were represented as median and quartile 1 and quartile 3. The chi–square test was used to calculate the Hardy–Weinberg equilibrium. The difference between the two groups with non-normally distributed variables was determined using the Mann–Whitney test. The variables that presented a statistically significant difference in univariate analysis were used in the multivariate logistic regression. A graphical representation of the logistic regression predicted probabilities was created, using an area under the receiver operating characteristics (AUROC) curve. A *p* value under 0.05 was considered statistically significant.

## 3. Results

Out of the 167 patients, 55 had isolated distal vein thrombosis and 112 had either proximal or proximal and concomitant distal vein thrombosis. The latter patients will be further referred to as having proximal DVT, because of the similar risk of pulmonary embolism affiliated with the two joined conditions.

In the univariate analysis, the conditions associated more frequently with proximal DVT were: male gender, neoplasms, previous DVT, and the presence factor V Leiden (Table 1). Bed rest was associated more frequently with distal DVT.

In the multivariate logistic regression (Table 2), gender maintained statistical significance with males being 2.43 times more likely than females to present proximal DVT. The neoplasia did not maintain statistical significance. Patients who presented previous DVT were 3.04 times more likely to have proximal DVT compared to patients that did not have previous DVT. The factor V Leiden was associated with proximal DVT (OR, 2.4). Bed rest was independently associated with isolated distal DVT (OR, 0.24).

**Figure 1:**
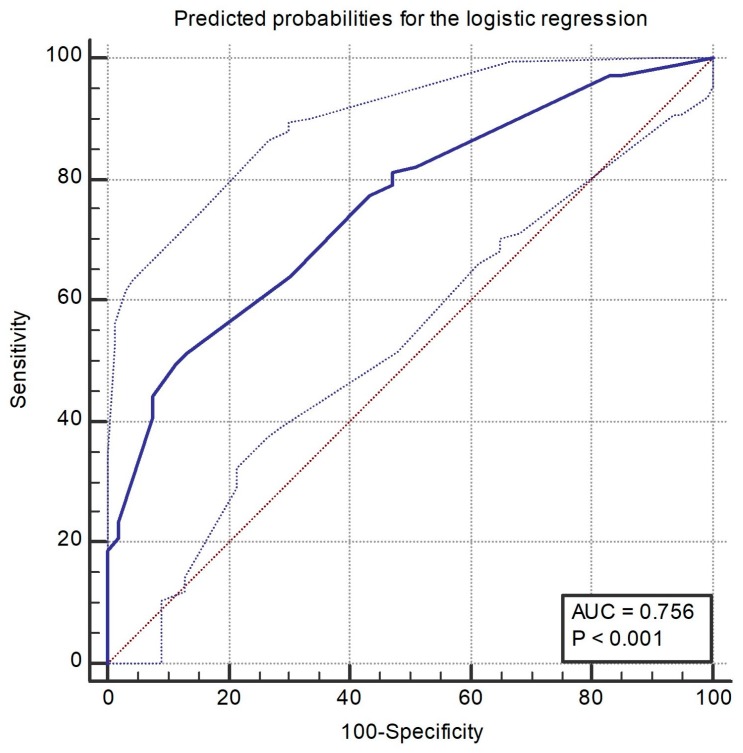
Area under the receiver operating characteristics (AUROC) curve for predicted probabilities for the logistic regression.

We used the predicted probabilities from the logistic regression to create an AUROC curve (Figure 1).

## 4. Discussions

The present study evaluated the probability of several parameters being associated with a specific DVT localization, either proximal or distal.

It is well known that men have a higher chance of DVT and PTE compared to women even when adjusting for the reproductive-related risks presented by women, like the intake of contraceptive pills [9,10,26]. Aside from this, in recent years the research community has manifested a growing interest regarding the differing presentation of DVT in men and women [26]. Trinchero et al., as well as Bauersachs et al., have shown the influence of gender on the incidence of proximal and distal DVT, with men having a higher chance of proximal DVT [11,27]. Barco et al. have shown differences in first presentation between men and women, with women generally presenting to the doctor following the early symptoms of distal DVT [23]. In our study, similarly to the aforementioned studies, we have observed a higher prevalence of proximal DVT in men, compared to women. Another explanation proposed by the literature is that women aged between 18 and 50 years are exposed to minor risk factors like oral contraception and pregnancy, which were linked to DVT incidence and specific locations of thrombosis [12]. However, as we excluded women with reproductive risk factors, there must be other factors accountable for the increased chance of proximal DVT in men. This premise sets the background for future research studies well worth pursuing.

Neoplasms are known to produce a hypercoagulability state, which increases the risk of thrombosis. This is especially true for lung, stomach and pancreas cancer and adenocarcinomas of unknown primary site, which are most commonly associated with DVT. Based on this correlation, DVT was reported to arise as a result of a paraneoplastic syndrome, revealing in some cases an occult cancer [28,29]. Nevertheless, in the present study, neoplasms presented an association with proximal DVT only in the univariate analysis. This might be due to the relatively small number of oncologic patients in our cohort. Thus, further studies with a better characterization of the specific neoplasms and more oncologic patients are needed to formulate a conclusion in this regard.

It is noteworthy that patients with previous DVT were observed to present more frequently with a proximal localization of DVT. This remained statistically significant in the multivariate model and may show a predisposing terrain to developing DVT. The predisposing terrain can also be influenced more or less markedly by genetic factors with a role in coagulation. Most commonly, these are either SNPs or mutations in gene encoding elements of the coagulation pathway, in factors like Leiden, prothrombin, PAI or in enzymes related to oxidative stress, such as MTHFR for hypercoagulability, or in factors VIII, IX (involved in hypocoagulability) [13,30,31]. In the case of somatic mutations, it has also been shown that the allelic burden of those mutations has an influence on thrombotic episodes, especially in myeloproliferative neoplasms [32]. In the present study, out of all the inventoried genetic factors, only mutant factor V Leiden was associated with proximal DVT in the univariate analysis, and it maintained statistical significance in the multivariate analysis.

We report that bed rest reduced the incidence of proximal DVT in the multivariate analysis, and it was observed to associate more commonly with distal DVT. Thus, this variable, which promotes venosus stasis, is more likely to to predispose to a distal DVT [33,34,35]. Our findings are noteworthy, since outlining a detailed risk factor profile for proximal DVT and PE in the general population could potentially lead to a tailored, personalized approach in the prophylaxis and therapy of proximal DVT, thus lowering DVT and PE mortality and morbidity.

## 5. Conclusions

Male sex, previous DVT and factor V Leiden mutation were independently associated with proximal DVT, while bed rest was independently associated with distal DVT. Thus, factors acting systemically elicit the occurrence of proximal DVT, while factors acting locally predispose to distal DVT. Future research focusing on refining the risk factor profile for proximal DVT and PE, that could design a personalized strategy in the prophylaxis and therapy of proximal DVT, is needed.

## Figures and Tables

**Table 1:** Comparison between patients with distal and proximal deep vein thrombosis (DVT).

		Isolated Distal DVT	Proximal DVT	*p*
		*n* = 55	*n* = 112	
Age (years)		67 (56; 74)	62 (53; 71)	0.4
Superficial vein thrombosis (SVT)	Yes	24 (44%)	37 (33%)	0.2
	No	31 (56%)	75 (67%)	
Gender	Female	35 (64%)	48 (43%)	0.01
	Male	20 (36%)	64 (57%)	
Place of origin	Rural	20 (36%)	40 (36%)	1
	Urban	35 (64%)	72 (64%)	
Neoplasia	Yes	2 (4%)	17 (15%)	0.03
	No	53 (96%)	95 (85%)	
Chemotherapy	Yes	1 (2%)	7 (6%)	0.2
	No	54 (98%)	105 (94%)	
Diabetes	Yes	6 (11%)	21 (19%)	0.2
	No	49 (89%)	91 (81%)	
Heart failure	Yes	8 (15%)	12 (11%)	0.4
	No	47 (85%)	100 (89%)	
Previous DVT	Yes	15 (27%)	61 (54%)	<0.001
	No	40 (73%)	51 (46%)	
Obesity (BMI > 30 kg/cm^2^)	Yes	15 (27%)	28 (25%)	0.8
	No	40 (73%)	84 (75%)	
Previous pulmonary thromboembolism (PTE)	Yes	0 (0%)	6 (5%)	0.1
	No	55 (100%)	106 (95%)	
Immobilization over 3 days	Yes	3 (5%)	2 (2%)	0.3
	No	52 (95%)	110 (98%)	
Bed rest	Yes	11 (20%)	7 (6%)	0.01
	No	44 (80%)	105 (94%)	
Car trip over 4 h	Yes	0 (0%)	4 (4%)	0.3
	No	55 (100%)	108 (96%)	
Major surgery in the last month	Yes	3 (5%)	7 (6%)	1
	No	52 (95%)	105 (94%)	
Varicose veins	Yes	31 (56%)	54 (48%)	0.3
	No	24 (44%)	58 (52%)	
Infection	Yes	10 (18%)	12 (11%)	0.2
	No	45 (82%)	100 (89%)	
Vasculitis	Yes	1 (2%)	3 (3%)	1
	No	54 (98%)	109 (97%)	
Fracture	Yes	2 (4%)	3 (3%)	0.664
	No	53 (96%)	109 (97%)	
Local trauma	Yes	2 (4%)	4 (4%)	1
	No	53 (96%)	108 (96%)	
PTE	Yes	1 (2%)	7 (6%)	0.273
	No	54 (98%)	105 (94%)	
Factor V Leiden	AA	0 (0%)	3 (3%)	0.05
	GA	6 (11%)	28 (25%)	
	GG	47 (85%)	80 (72%)	
Factor V Leiden	GA + AA	6 (11%)	31 (28%)	0.01
	GG	47 (85%)	80 (72%)	
Factor V Leiden Allele	G	100 (94%)	188 (85%)	0.01
	A	6 (6%)	34 (15%)	
Prothrombin	GA	3 (6%)	8 (7%)	1
	GG	51 (94%)	104 (93%)	
Prothrombin allele	G	105 (97%)	216 (97%)	1
	A	3 (3%)	8 (3%)	
PAI	4G/4G	14 (26%)	37 (33%)	0.2
	4G/5G	21 (39%)	49 (44%)	
	5G/5G	19 (35%)	25 (23%)	
PAI	4G	49 (45%)	123 (55%)	0.1
	5G	59 (55%)	99 (45%)	
MTHFR C677T	TT	9 (17%)	15 (14%)	0.06
	CT	29 (56%)	41 (39%)	
	CC	14 (27%)	49 (44%)	
MTHFR 677 Allele	C	57 (55%)	139 (66%)	0.06
	T	47 (45%)	71 (34%)	
MTHFR A1298C	CC	1 (2%)	12 (11%)	0.1
	AC	26 (49%)	50 (47%)	
	AA	26 (49%)	44 (42%)	
MTHFR 1298 Allele	A	78 (74%)	138 (65%)	0.1
	C	28 (26%)	74 (35%)	

**Table 2:** Multivariate analysis for the location of DVT.

	B	P	OR	95% CI	
				Lower	Upper
Male gender	0.89	0.02	2.43	1.13	5.23
Neoplasia	1.48	0.06	4.43	0.92	21.19
Previous DVT	1.11	0.005	3.04	1.40	6.60
Bed rest	−1.45	0.01	0.23	0.07	0.74
Factor V Leiden (GA+AA)	0.87	0.05	2.40	0.8	6.72
